# Effect of dietary calcium concentration and exogenous phytase on inositol phosphate degradation, mineral digestibility, and gut microbiota in growing pigs

**DOI:** 10.1093/jas/skad254

**Published:** 2023-08-01

**Authors:** Nicolas Klein, Naomi Sarpong, Tanja Melzer, Dieter Feuerstein, Charlotte M E Heyer, Amélia Camarinha-Silva, Markus Rodehutscord

**Affiliations:** Institute of Animal Science, University of Hohenheim, 70599 Stuttgart, Germany; Institute of Animal Science, University of Hohenheim, 70599 Stuttgart, Germany; Core Facility Hohenheim, University of Hohenheim, 70599 Stuttgart, Germany; BASF SE, 67063 Ludwigshafen, Germany; Institute of Animal Science, University of Hohenheim, 70599 Stuttgart, Germany; Institute of Animal Science, University of Hohenheim, 70599 Stuttgart, Germany; Institute of Animal Science, University of Hohenheim, 70599 Stuttgart, Germany

**Keywords:** calcium, exogenous phytase, inositol phosphate, microbiota, phosphorus, pig

## Abstract

Variations in the dietary Ca concentration may affect inositol phosphate (**InsP**) degradation, and thereby, P digestibility in pigs. This study assessed the effects of dietary Ca concentration and exogenous phytase on InsP degradation, nutrient digestion and retention, blood metabolites, and microbiota composition in growing pigs with ileal cannulation. In a completely randomized row–column design with four periods, eight ileal-cannulated barrows (initial body weight 27 kg) were fed four corn–soybean- and rapeseed meal-based diets containing 5.5 or 8.5 g Ca/kg dry matter (**DM**), with or without 1,500 FTU of an exogenous hybrid-6-phytase/kg diet. No mineral P was added and the P concentration in the feed was 4.8 g P/kg DM. Prececal InsP_6_ disappearance in pigs fed diets containing exogenous phytase was lower (*P* = 0.022) with additional Ca than without. Concentrations of InsP_2-4_ isomers and *myo*-inositol in the distal ileal digesta and prececal P digestibility were greater (*P* < 0.001) with exogenous phytase than without exogenous phytase. In feces, InsP_6_ disappearance was lower (*P* < 0.002) and concentration of InsP_5_ and InsP_4_ isomers was higher (*P* ≤ 0.031) with additional Ca compared to without additional Ca. The prececal amino acid digestibility, energy digestibility, and hindgut disappearance of energy did not differ. The Shannon diversity index of the microbiota in the distal ileal digesta and feces was similar among the diets but was lower in the distal ileal digesta than in the feces (*P* < 0.001). Permutation analysis of variance revealed no dietary differences between the bacterial groups within the ileal digesta and fecal samples (*P* > 0.05). In conclusion, additional Ca reduced the effect of exogenous phytase on prececal InsP_6_ degradation. Endogenous InsP degradation was impaired by additional Ca only in the hindgut but the abundance of bacterial genera in feces was not affected.

## Teaser

Increasing the dietary calcium concentration reduces the effects of exogenous phytase on prececal phytate degradation and endogenous phytate degradation in the hindgut of growing pigs.

## Introduction

Phytate is the salt of phytic acid (*myo*-inositol 1,2,3,4,5,6-hexakis [dihydrogen phosphate]s; **InsP**_**6**_) and is the main storage form of phosphorus (**P**) in plant feed ingredients (Eeckhout and Paepe, 1994). The release of InsP_6_–P depends on exogenous and plant intrinsic phytases in the feed because intestinal endogenous phytase activity is very low in pigs (Hu et al., 1996). However, InsP_6_ disappearance from the digestive tract may be reduced by the addition of Ca for different reasons. First, with more Ca in the diet, more Ca is available in the gastrointestinal tract for complexing with InsP_6_, whereby InsP_6_ hydrolysis progresses less readily (Wise and Gilburt, 1981). Second, dietary Ca concentration generally varies with limestone addition, which is associated with a high acid binding capacity as it takes 15 ± 2 mEq of 0.1 M HCl to lower 1 g of limestone to pH 3 (Lawlor et al., 2005). This is crucial, as Ca–InsP_6_ complexes are considered insoluble at an intestinal pH greater than 5 (Selle et al., 2009), thus, chelated InsP_6_ is no longer available for hydrolysis by phytase. Lastly, surplus Ca^2+^ ions may compete directly with the active site of the phytase (Qian et al., 1996).

The concentrations of Ca and P in the feed may also affect the microbial composition and bacterial activity in the gastrointestinal tract of pigs (Metzler-Zebeli et al., 2011, 2013). However, few studies have examined the interactions between dietary Ca concentration and exogenous phytase in the gastrointestinal tract of pigs. Hu et al. (2023) observed an effect of Ca on InsP_6_ disappearance, irrespective of the presence of exogenous phytase, whereas Sandberg et al. (1993) observed no effect of dietary Ca on InsP_6_ disappearance by the end of the ileum, but rather, in the feces when diets without exogenous phytase were fed.

Therefore, the objectives of this study were to investigate the effects of dietary Ca concentration and exogenous phytase on 1) InsP degradation, nutrient digestibility and retention, blood metabolites, and 2) microbial composition in the ileal digesta and feces of pigs, as well as the concentration of volatile fatty acids (**VFA**) in the feces. We hypothesized that the effects of exogenous phytase on InsP degradation, nutrient digestibility, and microbial groups in the digesta and feces are dependent on the Ca concentration of the feed.

## Materials and Methods

The experiment was approved by the Regierungspräsidium Stuttgart, Germany, in agreement with the German Animal Welfare Regulations (approval No. 35-9185.81/0494). The care of the animals throughout the trial was in accordance with Directive 2010/63/EU (European Parliament and the Council of the European Union, 2010).

### Experimental diets

The experimental diets followed a 2 × 2 factorial arrangement with two dietary Ca concentrations achieved by limestone addition (6 and 14 g/kg diet) and two inclusion levels of exogenous 6-phytase (0 and 1,500 FTU/kg diet, added on top of the mix; Table 1). A hybrid-6-phytase (Natuphos E; Rychen et al., 2017) was used. The inclusion of 6 g limestone/kg diet targeted a Ca:digestible P ratio of 2:1 in the diet (assuming a P digestibility of 60% when exogenous phytase is present; Gesellschaft für Ernährungsphysiologie (GfE, 2008), whereas the inclusion of 14 g limestone/kg diet was chosen to target a Ca:digestible P ratio of 3:1. The inclusion of 8 g/kg diet of diatomaceous earth balanced the mass differences between the dietary Ca concentrations. All the diets were based on corn, soybean meal, and rapeseed meal. Titanium dioxide was used as an indigestible marker. Mineral P was not added to the diets.

**Table 1.** Ingredient composition of the experimental diets

Dietary Ca, g/kg DM	5.5				8.5		
Exogenous phytase, FTU/kg of diet	0		1,500		0		1,500
Ingredients, g/kg as-fed							
Corn^1^				591			
Soybean meal^1^				250			
Rapeseed meal^1^				100			
Soybean oil				20			
P-free mineral and vitamin premix^2^				20			
Titanium dioxide				5			
Limestone		6				14	
Diamol^3^		8				—	
Exogenous phytase, FTU/kg of diet	0		1,500		0		1,500

^1^Analyzed concentration of nutrients is described in Supplementary Table S1.

^2^BASU Mineralfutter GmbH, Bad Sulza, Germany; provided the following per kilogram of diet: Ca, 0.88 g; Na, 1.0 g; Mg, 200 mg; Fe, 80 mg (iron sulfate); Mn, 50 mg (manganese oxide and sulfate); Zn, 60 mg (zinc oxide and sulfate); Cu, 10 mg (copper sulfate); I, 1.34 mg (calcium iodate); Se, 0.26 mg (sodium selenite); vitamin A, 7,000 IU; vitamin D, 1,000 IU; vitamin E; 80 mg; vitamin K, 1.0 mg; vitamin B_1_, 1.0 mg; vitamin B_2_, 3.1 mg; vitamin B_6_, 2.5 mg; vitamin B_12_, 20µg; niacin, 12.5 mg; pantothenic acid, 8.0 mg; folic acid, 0.4 mg; biotin, 0.08 mg; choline chlorides, 160 mg.

^3^Provided by BASU Mineralfutter GmbH, Bad Sulza, Germany.

### Animals, experimental design, sample collection, and preparation

Eight barrows (initial BW 27.3 ± 1.1 kg; final BW 59.0 ± 2.4 kg; German Landrace × Piétrain) were obtained from the Agricultural Research Station, Unterer Lindenhof (Eningen unter Achalm, Germany) of the University of Hohenheim. They were kept individually in stainless steel metabolism units (1.5 × 0.8 × 1.0 m). Room temperature was controlled at 22.0 ± 0.5 °C. The pigs were surgically fitted with a simple T-cannula in the distal ileum (Li et al., 1993). Pigs were fed three times the estimated energy requirement for maintenance (440 kJ ME/kg^0.75^ BW; GfE, 2008) per day, provided in two equal meals at 0715 and 1915 hours. The feed was mixed with water (1:1; w:w) immediately before feeding and ingested within 15 min. Feed residues in the trough were rare and were collected and frozen for subsequent analysis.

The experiment was arranged in a completely randomized row–column design with eight pigs, four diets, and four periods to achieve eight replicates per diet. The experimental periods of 12 d each consisted of a 5-d dietary adaption, followed by a 4-d feces and urine collection, 2 d of ileal digesta collection, and 1 d of blood collection. Feces were collected from the floor by hand immediately after defecation. Urine was collected by placing a clean bucket under the pig as soon as the pig started urinating. Two persons were present in the barn during day time to ensure the start of urination was noticed. A subsample of each defecation was taken with a sterile ­spatula for microbiota analysis and was immediately frozen at −18 °C. Ileal digesta were collected between 0715 and 1915 hours by attaching plastic bags to the open barrel of the cannula with rubber bands, as described by Rosenfelder-Kuon et al. (2020). A subsample of each filled plastic bag was taken by pouring a few milliliters of ileal digesta into a sterile container for microbiota analysis. Samples were frozen immediately at −18 °C. On the last day of each period, a blood sample was obtained from each pig via venipuncture 4 h after the morning meal, as described by Klein et al. (2021).

The ileal digesta, feces, and urine subsamples were pooled for each pig and period. The ileal digesta and feces were lyophilized (Delta 1-24 LSC, Martin Christ Gefriertrocknungsanlagen, Osterode am Harz, Germany). Ingredients, diets, lyophilized feces, and ileal digesta were ground through a 0.5-mm sieve (Ultra-Zentrifugalmühle ZM 200, Retsch, Haan, Germany) and pulverized using a vibrating cup mill (Pulverisette 9, Fritsch, Idar-Oberstein, Germany) or a mixer mill (MM 400, Retsch, Haan, Germany).

### Chemical analyses

Dry matter, crude nutrients, and gross energy (**GE**) were determined in the feed ingredients, diets, feces, and ileal digesta using standard assays (VDLUFA, 2007). Concentrations of total P, Ca, and Ti in diets, feces, and digesta, and total P and Ca in urine were analyzed using the modified sulfuric and nitric acid wet digestion method (Boguhn et al., 2009), followed by measurement using an inductively coupled plasma optical emission spectrometer (Vista Pro, Varian Inc., Australia). The concentrations of K in the diet, feces, and urine were analyzed accordingly, but measured at a wavelength of 766 nm. Inorganic P (**P**_**i**_) and Ca in blood serum were analyzed as described by Sommerfeld et al. (2018a). Activity of alkaline phosphatase (**ALP**) was determined by measuring the formation of p-nitrophenol from p-nitrophenyl phosphate in the presence of 2-amino-2-methyl-1-propanol. Blood urea nitrogen (**BUN**) was measured using a Beckman Olympus AU480 instrument (Beckman Coulter, Krefeld, Germany), based on an adaptation of the enzymatic method described by Talke and Schubert (1965). Blood analyses were conducted by IDEXX BioAnalytics (Kornwestheim, Germany).

The concentrations of InsP_6_ and InsP_3-5_ isomers in the feed ingredients, diets, feces, and digesta were determined according to the method of Zeller et al. (2015), with modifications as described by Sommerfeld et al. (2018b). For the extraction of InsP_1-2_ isomers, a buffer containing 50 mM Tris, 50 mM glycine, and 0.2 M sodium fluoride at a pH of 9 was used. All InsP were measured using high-pressure ion chromatography (ICS-3000 system, Dionex, Idstein, Germany). Because the Ins(1,2,6)P_3_, Ins(1,4,5)P_3_, and Ins(2,4,5)P_3_ isomers co-elute in this measurement, they were summarized as InsP_3x_. The results did not differentiate between the d- and l-forms of the measured InsP. *Myo*-inositol was determined using a gas chromatograph/mass spectrometer (7890B/5977A, Agilent Technologies, Waldbronn, Germany) as described by Sommerfeld et al. (2018b). The phytase activity of the feed was analyzed according to ISO EN 30024 (2009). Diets and ileal digesta were analyzed for amino acids (**AA**; Rodehutscord et al., 2004) using an L-8900 Amino Acid Analyzer (VWR, Hitachi, Tokyo, Japan), following sample oxidation and acid hydrolysis. The content of *N*-acetylneuraminic acid (**Neu5Ac**), a marker of mucin synthesis (Mesina et al., 2019), was determined using high performance anion exchange chromatography with pulsed amperometric detection. Mucin was extracted from ileal digesta samples with 0.15 M NaCl solution and cleaned up by repeated overnight precipitation with ethanol at −18 °C, following a protocol adapted from Lien et al. (1997). After clean-up, mucin was hydrolyzed to obtain Neu5Ac, according to Hurum and Rohrer (2011), by incubation for 3 h at 80 °C in 2 M acetic acid. The supernatant was filtered through a 0.2-µm polyamide syringe filter and diluted 1:25 for measurements. Neu5Ac measurements were conducted on a Dionex ICS 5000 + system (Thermo Fisher Scientific, Waltham, MA, USA), using a CarboPac MA1 column and an isocratic eluent of 250 mM sodium acetate in 100 mM sodium hydroxide. Quantification was performed using an external five-point-calibration of Neu5Ac standards at concentrations between 0.1 and 10 mg/L. The VFA concentrations in the feces were determined using ultrapure water-diluted samples by using vacuum distillation and gas chromatography (Hewlett-Packard 6890; Agilent Technologies), as described by Wischer et al. (2013).

### DNA extraction, illumina amplicon sequencing, and data analysis

The DNA was extracted from 0.25 g of each ileal digesta and fecal sample using FastDNA SPIN Kit for Soil (MP Biomedical, Solon, OH, USA) with some adjustments (Burbach et al., 2016). The DNA was quantified with a NanoDrop 2000 spectrophotometer (Thermo Fisher Scientific) and stored at −20 °C. Amplification of the V1 to V2 region of the 16S rRNA gene was performed according to the method described by Kaewtapee et al. (2017). Amplicons were verified by agarose gel electrophoresis, purified, and normalized using the SequalPrep Normalization Kit (Invitrogen, Carlsbad, CA, USA). Samples were sequenced using 250 bp paired-end sequencing chemistry on an Illumina NovaSeq 6000 (Illumina Solutions, Berlin, Germany).

### Calculations

Prececal (**pc**) InsP_6_ disappearance and digestibilities of nutrients and GE were calculated as follows:


$$\begin{array}{l} y(X)=100-100\times \left(\frac{Ti\;in\;diet}{Ti\;in\;digesta}\right)\times \left(\frac{X\;in\;digesta}{X\;in\;diet}\right) \end{array}$$ (1)

where *y*(*X*) is the disappearance or digestibility of *X* (%) and *X* is the concentration of InsP_6_, DM, nitrogen (**N**), AA, Ca, P (g/kg), or GE (MJ/kg). The corresponding values for the total digestive tract were calculated using the fecal concentrations.

Hindgut disappearance of InsP_6_, P, Ca, and N (g/kg) was calculated as follows:


$$\begin{array}{l} y(X)=X_{TT}-X_{pc} \end{array}$$ (2)

where *y*(*X*) is the hindgut disappearance of *X* in g/kg or MJ/kg of feed, and *X*_TT_ and *X*_pc_ are the total tract or pc disappearance of N, Ca, P, InsP_6_ (g/kg) and GE (MJ/kg) of feed.

The amount of excreted urine (kg/d) was calculated as follows:


$$\begin{array}{l} excreted\;urine=\frac{K_{TT}-K_{A}\;}{K_{U}} \end{array}$$ (3)

where *K*_TT_ is the total tract digested amount of K in g/d; *K*_A_ is the assumed accretion of K by the pig in g/d, according to GfE (2008) (1.9 g/kg BW gain) and *K*_U_ is the analyzed K concentration in the urine in g/kg. It was assumed that the difference between *K*_TT_ and *K*_A_ must represent the amount of K excreted via the urine.

The urinary excretion of P, Ca, *myo*-inositol, and N was calculated as the product of the measured concentration in the urine and the amount of urine calculated by Equation 3. The retained quantities of Ca and P were calculated as the difference between the intake and excretion of the respective elements in the feces and urine.

### Statistical analysis

Data were analyzed using a 2 × 2 factorial analysis of variance using the MIXED procedure in SAS (version 9.3; SAS Institute Inc., Cary, NC, USA). The model was


$$\begin{array}{l} y_{ijkl}=\mu +a_{i}+b_{j}+{\left(ab\right)}_{ij}+c_{k}+\tau_{l}+e_{ijkl} \end{array}$$ (4)

where *y*_*ijkl*_ is the response variable, µ is the overall mean, *a* is the fixed effect of dietary Ca concentration (*i* = 5.5 or 8.5 g/kg DM), *b* is the fixed effect of exogenous phytase inclusion (*j* = 0 or 1,500 FTU/kg), (*ab*)_*ij*_ is the fixed interaction effect between the *i*th dietary Ca concentration and the *j*th exogenous phytase inclusion, *c* is the random effect of the animal (*k* = 1 to 8), τ is the random effect of the period (*l* = 1 to 4), and *e*_*ijkl*_ is the residual error.

The InsP_6_ disappearance and nutrient digestibility data were logit-transformed, and concentrations in the digesta, feces, urine, and blood were log- or square-root-transformed to meet the assumptions of variance homogeneity and normality. If the *F*-test results were significant, a multiple *t*-test was used for pairwise treatment comparisons. All results are presented via a letter-based display as the least-squares means of the untransformed data. Statistical significance was set at *P* ≤ 0.05.

Bioinformatics assembly and alignment of raw sequencing reads were performed using the MOTHUR pipeline (Kozich et al., 2013). UCHIME was used to identify possible chimeras (Edgar et al., 2011). The amplicon sequence variants (**ASV**) were identified using the SILVA database (Release 138.1). For unclassified ASV, the nearest representative was manually detected using seqmatch from the Ribosomal Database Project (Cole et al., 2014). Only ASV with an average relative abundance > 0.0001% and >250 bp were used for further analyses. One fecal sample was excluded from further analysis because of a lower number of reads. The Shannon diversity index was calculated to evaluate bacterial diversity within the samples using the *vegan* package in R (version 4.0.0; 2020-04-24).

After the sequencing reads were standardized using total reads, a sample dissimilarity matrix was constructed using the Bray–Curtis dissimilarity coefficient. This was subjected to a permutation analysis of variance (**PERMANOVA**) using the *Adonis* function to compare the microbial communities associated with diets within sample types (digesta and feces) as well as between digesta and fecal samples.

To visualize the ordination and clustering of the samples, nonmetric multidimensional scaling (**NMDS**) was plotted using the R package, *ggplot2*. The Wilcoxon test followed by the Benjamini–Hochberg procedure was conducted to analyze differences in abundance at the phylum and genus levels between both sample types. Spearman’s correlation coefficients between fecal genera with an average relative abundance > 1% and items from hindgut disappearance, total tract digestibility, and VFA concentrations were calculated after the determination of the *z*-scores for standardization. Visualization was performed using the R package, *svglite*. Statistical significance for all results was set at *P* ≤ 0.05. Sequences were submitted to the European Nucleotide Archive under the accession number, PRJEB61474.

## Results

The Ca concentration ranged from 5.4 g/kg DM in the diets without additional Ca to 8.4 and 8.6 g/kg DM in diets with additional Ca (Table 2). The analyzed total P concentration was 4.8 g/kg DM in all diets, whereof 3 g was bound in InsP_5-6_ provided by corn (45%), followed by soybean (30%), and rapeseed meal (25%; Supplementary Table S1). The results confirmed that intrinsic plant phytase activity in the diets without phytase was not detectable (<50 FTU/kg diet), and phytase activity in diets with exogenous phytase ranged from 1,470 to 1,620 FTU/kg. Analyzed AA concentrations of the diets are shown in Supplementary Table S2 and exceeded the formulated values of indispensable AA between 0.6 g/kg DM (Met) and 3.6 g/kg DM (Leu).

**Table 2.** Analyzed chemical composition of the experimental diets (g/kg DM unless otherwise stated)

Dietary Ca, g/kg DM	5.5		8.5	
Exogenous phytase, FTU/kg of diet	0	1,500	0	1,500
DM, g/kg	896	895	900	897
GE, MJ/kg DM	19.2	19.1	19.0	19.0
CP (nitrogen × 6.25)	229	222	220	222
Ether extract	34	33	34	34
aNDFom^1^	128	130	127	125
ADFom^2^	58	60	61	59
ADL	20	17	18	15
Crude fiber	36	37	37	37
Crude ash	73	75	69	72
Calcium	5.4	5.4	8.6	8.4
Total phosphorus (P)	4.8	4.8	4.8	4.8
InsP_6_–P	2.9	2.9	2.8	2.7
InsP_6_, µmol/g DM^3^	15.5	15.5	14.9	14.8
Ins(1,2,4,5,6)P_5_, µmol/g DM	1.1	1.1	1.0	1.0
Ins(1,2,3,4,5)P_5_, µmol/g DM	0.6	0.5	0.6	0.6
*Myo*-inositol, µmol/g DM	1.1	1.1	1.1	1.1
Phytase activity, FTU/kg	<50	1,470	<50	1,620

^1^aNDFom, neutral detergent fiber assayed with heat-stable amylase and expressed exclusive of residual ash.

^2^ADFom, acid detergent fiber expressed exclusive of residual ash.

^3^Inositol phosphate (InsP) isomers not mentioned in the table were not detectable or below limit of quantification.

The pc InsP_6_ disappearance was increased with supplemental phytase, but this increase was lower with additional Ca than without additional Ca (22.7% to 84.8% vs. 21.9% to 89.6%), implying a interaction (*P* < 0.022; Table 3). Total tract InsP_6_ disappearance was lower (*P* = 0.002) with additional Ca than without additional Ca and higher (*P* = 0.023) with supplemented phytase than without it. The hindgut disappearance of InsP_6_ was lower with additional Ca in diets without added phytase, but not in diets with added phytase (*P* = 0.045). The pc and total tract digestibility of P and Ca were greater (*P* < 0.001) with phytase than without phytase. Urinary P excretion was greater with phytase supplementation only in the diet without additional Ca (*P* < 0.001). Urinary Ca excretion was greater (*P* < 0.001) with additional Ca than without additional Ca, but lower (*P* < 0.001) with phytase than without phytase. The Ca retention was greater with additional Ca (*P* = 0.004) and phytase supplementation (*P* < 0.001) than without. The calculated disappearance of P and Ca from the hindgut did not differ. The digestibility and hindgut disappearance of GE also did not differ among the diets (Table 4). Total tract N digestibility was greater (*P* ≤ 0.019) with phytase than without phytase; however, N excretion in urine and N retention by pigs were similar. The pc digestibility of AA did not differ among the diets (Supplementary Table S3). Fecal VFA concentrations did not differ among the diets, except for isobutyric acid and isovaleric acid, which were greater with additional Ca (*P *≤ 0.037; Supplementary Table S4).

**Table 3.** Prececal, total tract, and hindgut InsP_6_ disappearance, prececal and total tract digestibility, hindgut disappearance (HD), and retention of P and Ca by growing pigs^1^

Dietary Ca, g/kg DM	5.5		8.5		SEM	*P*-value		
Exogenous phytase, FTU/kg of diet	0	1,500	0	1,500		Ca	Phytase	Ca × phytase
InsP_6_								
Prececal disappearance, %	21.9^c^	89.6^a^	22.7^c^	84.8^b^	1.42	0.068	<0.001	0.022
Total tract disappearance, %	95.9	98.5	87.0	96.0	3.34	0.002	0.023	0.988
HD, µmol/g DM	11.5^a^	1.4^c^	9.6^b^	1.7^c^	0.59	0.368	<0.001	0.045
P								
Prececal digestibility, %	22.9^c^	63.5^a^	25.9^b^	60.1^a^	1.37	0.840	<0.001	0.009
Total tract digestibility, %	28.2	61.2	26.3	58.6	1.51	0.086	<0.001	0.906
HD, g/kg DM	0.3	-0.1	0.0	-0.1	0.07	0.164	0.101	0.621
Intake, g/d	6.5	6.5	6.5	6.4	0.83	—	—	—
In feces, g/d	4.6	2.5	4.8	2.7	0.47	0.054	<0.001	0.792
In urine, mg/d	17^b^	355^a^	16^b^	18^b^	54	<0.001	<0.001	<0.001
Retention, g/d	1.8	3.6	1.6	3.8	0.37	0.703	<0.001	0.134
Ca								
Prececal digestibility, %	48.9^c^	72.0^a^	49.6^c^	64.2^b^	2.13	0.017	<0.001	0.007
Total tract digestibility, %	47.3^c^	71.2^a^	46.9^c^	63.9^b^	2.62	0.024	<0.001	0.038
HD, g/kg DM	-0.1	0.0	-0.2	0.0	0.10	0.662	0.166	0.645
Intake, g/d	7.3	7.3	11.6	11.4	1.21	<0.001	0.548	0.593
In feces, g/d	3.9	2.1	6.3	4.1	0.66	<0.001	<0.001	0.055
In urine, g/d	1.6^b^	0.3^c^	3.0^a^	1.7^b^	0.21	<0.001	<0.001	0.002
Retention, g/d	1.8	4.9	2.3	5.6	0.53	0.004	<0.001	0.650
Urinary *myo*-inositol excretion, mg/d	23	38	23	60	15	0.004	<0.001	0.440

^1^Least squares means based on eight observations per diet.

^a,b,c^Within a row, individual treatment means without a common superscript differ significantly (*P* < 0.05).

**Table 4.** Prececal and total tract digestibility, calculated hindgut disappearance (HD) of GE and N, and N retention by growing pigs^1^

Dietary Ca, g/kg DM	5.5		8.5		SEM	*P*-value		
Exogenous phytase, FTU/kg of diet	0	1,500	0	1,500		Ca	Phytase	Ca × phytase
GE								
Prececal digestibility, %	68.7	69.1	69.5	69.7	0.62	0.236	0.618	0.953
Total tract digestibility, %	84.6	84.9	85.1	85.4	0.38	0.079	0.236	0.999
HD, MJ/kg DM	3.0	3.0	3.0	3.0	0.12	0.569	0.903	0.790
N								
Prececal digestibility, %	76.0	75.9	75.1	76.2	0.68	0.617	0.448	0.377
Total tract digestibility, %	84.4	85.8	84.6	86.2	0.98	0.602	0.019	0.807
HD, g/kg DM	3.1	3.5	3.3	3.6	0.40	0.427	0.105	0.614
Intake, g/d	49.4	47.8	47.7	48.1	6.17	—	—	—
In feces, g/d	7.6	6.7	7.3	6.5	0.67	0.372	0.009	0.784
In urine, g/d	21.2	19.9	20.7	19.5	4.89	0.907	0.743	0.653
Retention, g/d	20.7	21.2	19.7	22.0	1.66	0.744	0.226	0.425

^1^Least squares means based on eight observations per diet.

In the ileal digesta, the concentrations of InsP_6_, Ins(1,2,3,4,5)P_5_, and Ins(1,2,4,5,6)P_5_ were lower (*P* < 0.001) with phytase, whereas the concentrations of the InsP_4_ isomers, InsP_3*x*_, and *myo*-inositol were higher (*P* < 0.001) than those without phytase (Table 5). Ins(1,2)P_2_ was only detected in the ileal digesta of pigs fed diets containing phytase, and its concentration was lower (*P* = 0.013) with the addition of Ca. In the feces, the concentrations of InsP_6_, Ins(1,2,4,5,6)P_5,_ and Ins(1,2,5,6)P_4_ were higher (*P* < 0.031) with additional Ca than without. Fecal concentrations of InsP_6_, Ins(1,2,4,5,6)P_5_, and *myo*-inositol were lower (*P* ≤ 0.015) with phytase than without. The Neu5Ac concentration in the ileal digesta was higher by a trend in pigs fed diets with additional Ca (*P* = 0.064) than without additional Ca. Urinary *myo*-inositol excretion was higher (*P* < 0.001) with phytase than without phytase and higher (*P* = 0.004) with additional Ca (Table 3).

**Table 5.** Concentrations of inositol phosphates (InsP) and *myo*-inositol in ileal digesta and feces of pigs^1^

Dietary Ca, g/kg DM	5.5		8.5		SEM	*P*-value		
Exogenous phytase, FTU/kg of diet	0	1,500	0	1,500		Calcium	Phytase	Calcium × phytase
Ileal digesta^2^, µmol/g DM								
InsP_6_	33.3^a^	4.5^c^	33.1^a^	6.6^b^	0.59	0.025	<0.001	0.023
Ins(1,2,4,5,6)P_5_	2.4	0.3	2.5	0.5	0.05	0.027	<0.001	0.078
Ins(1,2,3,4,5)P_5_	1.4	0.6	1.4	0.8	0.09	0.305	<0.001	0.516
Ins(1,2,3,4,6)P_5_	0.7	n.d.	0.7	n.d.	0.03	0.968	—	—
Ins(1,2,5,6)P_4_	0.5	1.9	0.4	2.3	0.23	0.646	<0.001	0.349
Ins(1,2,3,4)P_4_	0.3	2.3	0.3	2.9	0.31	0.389	<0.001	0.468
InsP_3x_^3^	0.5	8.1	0.4	8.1	0.59	0.880	<0.001	0.856
Ins(1,2)P_2_	n.d.	4.9	n.d.	2.9	0.63	0.013	—	—
* Myo*-inositol	1.5	7.0	1.5	7.0	0.76	0.748	<0.001	0.562
Neu5Ac^4^, g/kg DM	0.35	0.30	0.36	0.37	0.04	0.064	0.300	0.154
Feces^2^, µmol/g DM								
InsP_6_	3.7	1.4	11.6	3.8	2.89	0.004	0.015	0.487
Ins(1,2,4,5,6)P_5_	0.8	n.d.	1.3	0.3	0.21	0.013	<0.001	—
Ins(1,2,3,4,5)P_5_	n.d.	n.d.	0.4	0.2	0.15	—	0.361	—
Ins(1,2,5,6)P_4_	0.2	n.d.	0.3	n.d.	0.05	0.031	—	—
* Myo-*inositol	0.8	0.4	0.7	0.4	0.08	0.537	0.014	0.442

^1^Least squares means based on eight observations per diet.

^2^Inositol phosphate isomers not mentioned here were not detectable (n.d.) or below limit of quantification.

^3^A clear discrimination was not possible because of co-elution, at least one of the following isomers Ins(1,2,6)P_3_, Ins(1,4,5)P_3_, Ins(2,4,5)P_3_.

^4^ *N*-acetylneuraminic acid.

^a,b,c^Within a row, individual treatment means without a common superscript differ significantly (*P* < 0.05).

The serum P_i_ concentration was higher with added phytase than without, and lower with additional Ca in the diet without exogenous phytase, causing an interaction (*P* = 0.037; Table 6). The serum Ca concentration increased to a greater extent with additional Ca than with exogenous phytase (*P* < 0.001). The plasma *myo*-inositol concentration was increased by phytase supplementation (*P* < 0.001), whereas BUN concentration was reduced by phytase supplementation (*P* = 0.002). The serum ALP activity in the blood of one pig was consistently higher than the mean of the other pigs by a factor of 2.2. When the data of this pig were disregarded, the serum ALP activity decreased (*P *= 0.016) with the addition of phytase.

**Table 6.** Concentrations of inorganic P (P_i_), calcium (Ca), alkaline phosphatases (ALP), urea nitrogen (BUN), and *myo*-inositol in the blood of pigs^1^

Dietary Ca, g/kg DM	5.5		8.5		SEM	*P*-value		
Exogenous phytase, FTU/kg of diet	0	1,500	0	1,500		Calcium	Phytase	Calcium × phytase
P_i_, mmol/L	2.6^b^	3.4^a^	2.1^c^	3.2^a^	0.14	<0.001	<0.001	0.037
Ca, mmol/L	3.1^b^	3.0^c^	3.5^a^	3.1^b^	0.04	<0.001	<0.001	<0.001
ALP, U/L	253	232	237	224	34.67	0.950	0.051	0.767
ALP^2^, U/L	209	192	219	200	11.0	0.181	0.016	0.917
*Myo*-inositol, µg/mL	9.4	17.2	8.6	17.4	0.96	0.407	<0.001	0.499
BUN, mg/dL	21.5	17.9	19.6	18.3	2.07	0.269	0.002	0.065

^1^Least squares means based on eight observations per diet.

^2^Least squares means without one pig that had a 2.2-fold higher ALP activity than the mean of the other pigs.

^a,b,c^Within a row, individual treatment means without a common superscript differ significantly (*P* < 0.05).

**Figure 1.**
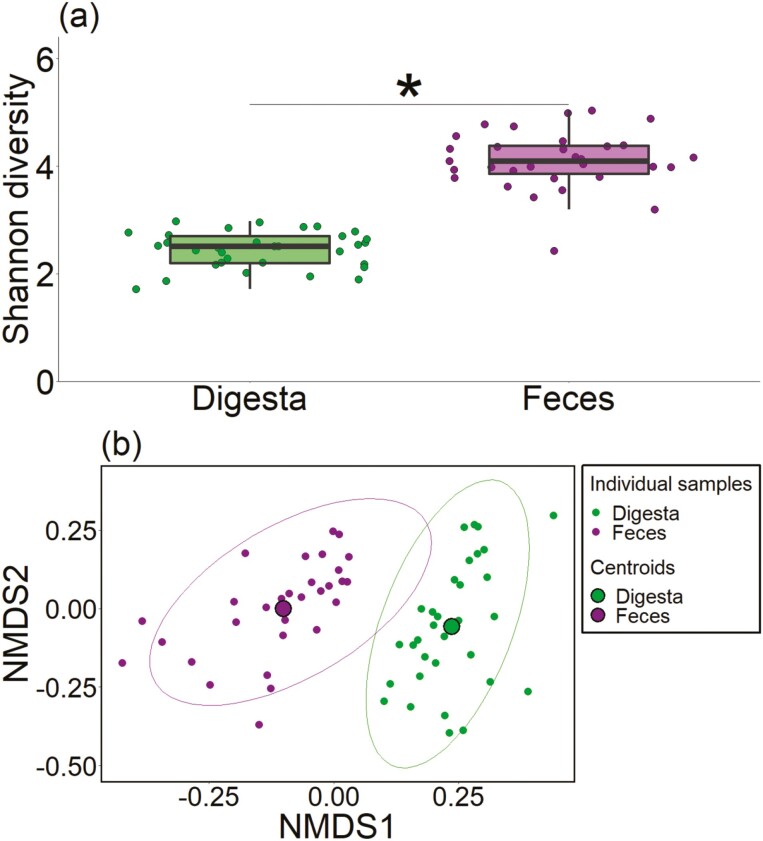
Comparison of the microbial community in the ileal digesta and fecal samples of pigs. (a) Shannon diversity index (*significant difference, *P* < 0.05), and (b) NMDS plot with centroids per group of samples.s

Amplicon sequencing of fecal and ileal digesta samples showed an average of 44,156 ± 3,213 reads per sample, and 2,648 ASV were identified from the reads. The Shannon diversity index in ileal digesta and fecal samples did not differ among the diets. However, the Shannon diversity index was lower in the ileal digesta than in the fecal samples (*P* < 0.001; Figure 1a). The PERMANOVA revealed no differences in the bacterial community of the ileal digesta among the diets (Supplementary Table S5) but showed a trend for the interaction of additional Ca × phytase in fecal samples (*P *= 0.09; Supplementary Table S6). Differences were found between the ileal digesta and fecal bacterial communities (*P* < 0.001; Supplementary Table S7), as indicated by the two distinct clusters in the NMDS plot and the distance between the two centroids (Figure 1b).

**Figure 2.**
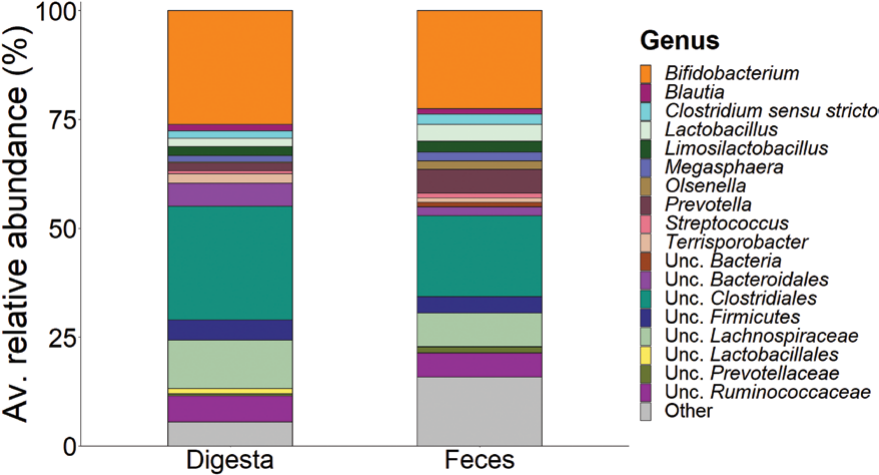
Comparison of the microbial community in the ileal digesta and fecal samples at genus level (‘Other’ includes genera with a relative abundance of <1%).

**Figure 3.**
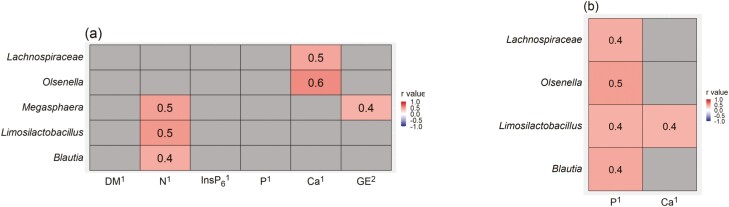
Significant correlations (*P* < 0.05) between bacteria in the feces (relative abundance > 1%), (a) hindgut disappearance, and (b) total tract digestibility (*P* < 0.05). ^1^g/kg DM, ^2^MJ/kg DM.

The most abundant phyla in both sample types were *Firmicutes* (ileal digesta, 64%; feces, 60.2%), followed by *Actinobacteria* (ileal digesta, 26.8%; feces, 26.2%), and *Bacteroidetes* (ileal digesta, 8.5%; feces, 11.0%). At the genus level, the most abundant group was *Bifidobacterium,* with no marked difference between ileal digesta and feces (Figure 2). *Terrisporobacter* was more abundant in ileal digesta than feces (*P* < 0.05; Supplementary Table S8), whereas *Lactobacillus* and *Limosilactobacillus* were less abundant in ileal digesta than feces (*P* ≤ 0.016). The relative abundance of *Megasphaera* (*P* = 0.012), *Limosilactobacillus* (*P *= 0.002), and *Blautia (P *= 0.021) were positively correlated with N hindgut disappearance (Figure 3a). *Megasphaera* abundance was positively correlated with GE hindgut disappearance (*P = *0.025). The relative abundance of unclassified Lachnospiraceae (*P* = 0.011) and *Olsenella* (*P* < 0.001) were positively correlated with the hindgut disappearance of Ca. Positive correlations with the total tract digestible P were detected for the relative abundance of *Olsenella* (*P* = 0.005), *Limosilactobacillus* (*P* = 0.016), *Blautia* (*P* = 0.098), and unclassified Lachnospiraceae (*P* = 0.014; Figure 3b). Most correlations found between fecal bacterial abundance and fecal VFA concentration were negative, except for *Prevotella* and *Bifidobacterium* (Supplementary Figure S1).

## Discussion

### Inositol phosphate degradation

In the present study, when the feed did not contain added phytase, pc InsP_6_ disappearance was not affected by the additional Ca. Variations in dietary Ca concentration from 3 to 11 g Ca/kg DM also did not affect pc InsP_6_ disappearance in pigs fed diets without exogenous phytase (Sandberg et al., 1993). Hu et al. (2023) detected a decrease in InsP_6_ disappearance when a basal diet without limestone (2 g Ca/kg DM) was added with limestone to a Ca concentration of 6 g Ca/kg DM, but not when further limestone was added to a Ca concentration of 10 g Ca/kg DM. In all these studies, pc InsP_6_ disappearance was low (≤35%) at the lowest Ca concentration in the feed. This might explain why a further reduction in pc InsP_6_ disappearance was hardly observed with the addition of Ca. The generally low pc InsP_6_ disappearance in pigs fed diets without phytase has often been reported in the literature (Rodehutscord et al., 2022) and is most likely related to low endogenous phytase activity in the small intestine (Hu et al., 1996; Yi and Kornegay, 1996). Phytase added to a diet with a low Ca concentration increased pc InsP_6_ disappearance to 90% in the present study, which confirmed the level found in phytase-supplemented diets in previous studies (Mesina et al., 2019; Lu et al., 2020; Rosenfelder-Kuon et al., 2020). A reduction in InsP_6_ disappearance by 5 percentage points in diets with phytase was found with additional Ca in the present study. Hu et al. (2023) also observed the reducing effect of additional Ca. The pH optima of the used phytase in both studies suggest a notable InsP_6_ degradation in the stomach (Greiner, 2021; Menezes-Blackburn et al., 2022). Insoluble Ca–InsP_6_ complexes precipitate at pH 5.4 (Kaufman and Kleinberg, 1971). Thus, most of the InsP_6_ might be hydrolyzed by the exogenous phytases in the stomach before insoluble complexes are formed at a decisive pH in the small intestine. Nevertheless, the addition of Ca by limestone increased the pH in the stomach from 3.8 to 4.1 (Hu et al., 2023). A shift in stomach pH away from the phytase optimum cannot be ruled out in the present study and may explain the reduction in InsP_6_ disappearance with additional Ca in the phytase-containing feed.

Consistent with previous studies, phytase addition in the present study increased the concentration of partially dephosphorylated InsP in the distal ileum digesta (Rosenfelder et al., 2020; Hu et al., 2023). The higher concentration of Ins(1,2,3,4,5)P_5_ compared to other InsP_5_ isomers in the presence of phytase confirmed its classification by Rychen et al. (2017) as a 6-phytase. The Ins(1,2,3,4,6)P_5_ isomer was detected only in the ileal digesta of pigs fed diets without phytase. Thus, mucosal phytase activity in pigs, although low overall, may include 5-phytase, as it has been previously suggested for laying hens (Sommerfeld et al., 2020a, 2020b) and gnotobiotic broiler chickens (Sommerfeld et al., 2019). Hybrid phytase used in the present study increased the ­concentrations of Ins(1,2,3,4)P_4_ and Ins(1,2,5,6)P_4_ in the distal ileum digesta, which is consistent with the results of other studies that used this phytase (Krieg et al., 2020). In studies that used an *Escherichia coli* 6-phytase, only Ins(1,2,5,6)P_4_ increased in the ileal digesta of pigs (Rosenfelder-Kuon et al., 2020). In contrast, the InsP_3x_ concentration increased upon phytase addition to a greater extent in the present study than in the study by Rosenfelder-Kuon et al. (2020). These differences in the distal ileum digesta indicate that the two 6-phytases differ in their degradation pathways. The concentration of Ins(1,2)P_2_ in the ileal digesta was lower with additional Ca in the phytase-supplemented diet. This is likely a consequence of the greater InsP_6_ concentration in the ileal digesta of pigs fed the respective diets. If the initial dephosphorylation step is impaired, less Ins(1,2)P_2_ is produced at the end of the ileum. This was consistent with the higher concentrations (although not significant) of all intermediate dephosphorylation products.

The *myo*-inositol concentration was markedly increased by adding exogenous phytase in ileum digesta and blood plasma of pigs. A similar effect has been reported in previous studies on pigs and poultry (Mesina et al., 2019; Lu et al., 2020; Klein et al., 2021; Novotny et al., 2023). Urinary *myo*-inositol excretion was also increased by phytase. Rats fed *myo*-inositol-deficient diets exhibited reduced urinary *myo*-inositol concentrations (Burton et al., 1976). The present study suggests that the increased release of *myo*-inositol caused by exogenous phytase might increase the overall *myo*-inositol pool in the animal, providing a plausible explanation for the observed increase in urinary and plasma *myo*-inositol levels. *Myo*-inositol excretion in urine was higher with additional Ca carbonate. However, the calculated *myo*-inositol excretion of one individual fed additional Ca carbonate with phytase was four times higher than the LSMean calculated for this diet (213 vs. 60.1 mg/d).

Results of fecal InsP_6_ disappearance indicated that microbial InsP_6_ breakdown in the large intestine was impaired by the additional Ca in the feed. This is consistent with the results of Sandberg et al. (1993) and might be attributed to Ca–InsP_6_ complexes formed along the gastrointestinal tract, which would remain insoluble in the large intestine because the physiological pH is above 6.0 (Merchant et al., 2011). The fecal concentrations of Ins(1,2,4,5,6)P_5_ and Ins(1,2,5,6)P_4_ were similarly affected but to a lesser extent than that of InsP_6_. The diminishing effect of Ca with each further hydrolysis step of InsP concurs with in vitro results of Kaufman and Kleinberg (1971). They observed a decreased capacity of lower InsP molecules to form insoluble Ca–InsP complexes under physiological pH conditions. The effects of Ca on fecal InsP concentrations were not related to variations in the relative abundance of any of the genera detected in this dataset. This might imply that the variable release of InsP_6_–P among the diets was not decisive for the abundance of bacteria in the large intestine.

### Mineral digestibility and mineral balance

In diets with phytase, Ca addition caused a reduction in pc digestible P by 0.2 g/kg DM (dietary P concentration multiplied by pc P digestibility). Although this effect was not significant, it reflects the reduction in pc InsP_6_–P disappearance, which was 0.3 g/kg DM (dietary InsP_6_–P concentration multiplied by InsP_6_ disappearance). Most of the difference of 0.1 g/kg DM may reflect P contained in partially degraded InsP and therefore remained indigestible to the pig (Rodehutscord and Bikker, 2022). Similarly, additional Ca did not affect the absorption of P released from InsP_6_, as observed by Hu et al. (2023).

Except for the diet without additional Ca and with phytase, the urinary excretion of P was approximately 17 mg P/d. This value was close to the estimated urinary P loss of 0.35 mg/kg BW/d (or 17.5 mg P/d for a pig with 50 kg BW), which was considered inevitable for growing pigs by Rodehutscord et al. (1998). This indicates that the absorbed P was completely utilized for P retention by the pigs in the present study, and P supply was the limiting factor for retention. However, in the diet without additional Ca and with phytase, urinary P excretion increased by a factor of 20. In addition, serum P_i_ concentration increased with this diet, indicating that the amount of Ca absorbed was insufficient to retain the P that became digestible upon phytase addition, although the Ca digestibility increased to 72% and Ca excretion in urine decreased to 0.3 g/d.

The Ca intake in diets with additional Ca increased by 4.2 g/d compared to diets without additional Ca, of which 2.2 and 1.4 g/d were excreted in feces and urine, respectively, leaving 0.5 to 0.7 g/d of Ca additionally retained compared to diets without additional Ca. This confirmed that absorption, endogenous secretion, and urinary excretion are involved in Ca homeostasis (Schröder and Breves, 2006). When phytase was added to the diet with additional Ca, a further increase in Ca retention of 0.7 g/d was observed compared to the phytase diet without additional Ca. Likewise, this reflects the effect of increased P digestibility and retention causing an increased metabolic demand of Ca for bone formation. Except for the treatment without phytase and without additional Ca, the ratio of Ca retention and P retention was in a narrow range of 1.36 to 1.47. Differences in serum Ca concentrations coincided with differences in urinary Ca excretion, which is consistent with the results of Schlegel and Gutzwiller (2017). The serum ALP activity was decreased with the addition of phytase, which is consistent with the study of Kiarie et al (2022), who observed a decrease in plasma ALP activity in nursery pigs with the addition of exogenous phytase to a low-P corn-based diet. The calculated hindgut disappearance of P and Ca did not differ, and the values were close to zero, indicating no net absorption of these minerals in the hindgut. Although the release of P from InsP_6_ in the hindgut was remarkable in diets without phytase, the released P was not absorbed, which is consistent with the results of previous studies (Schlemmer et al., 2001; Rutherford et al., 2014; Mesina et al., 2019; Rosenfelder-Kuon et al., 2020; Klein et al., 2021).

### Nitrogen balance, amino acid digestibility, and sialic acid

Pigs fed diets without phytase exhibited greater fecal N excretion. This suggests that when more P enters the hindgut, bacterial cell replication and metabolism increase, leading to increased N incorporation into microbial proteins, which are subsequently excreted (Metzler and Mosenthin, 2008). In addition to greater fecal N excretion, pigs fed diets without phytase showed higher BUN concentrations. Phosphate is involved in protein synthesis and insufficient digestible P intake may impair such processes, leading to higher AA catabolism and subsequent urea concentrations at the time of blood sampling. However, the quantitative urinary N excretion of pigs did not differ, indicating that any effect on the metabolic level was not generally relevant to N retention of the pigs. Additional Ca and phytase did not affect pc AA digestibility, indicating that AA was equally available for protein synthesis in all treatments. Mesina et al. (2019) rejected the hypothesis that dietary InsP_6_ could increase the endogenous secretion of proteins such as mucin, thereby increasing endogenous AA losses. Consistent with this, the Neu5Ac concentration in the distal ileum digesta, an indicator of mucin, was not significantly affected. The only indication of a trend was that mucin secretion may have been increased by the addition of Ca to the diet in the present study. In a meta-analysis of 34 publications, the pc AA digestibility was increased by an average of 0.9 (Met) to 1.5 percentage units (Thr; Zouaoui et al., 2018). However, phytase effects on AA digestibility were inconsistent among studies and overall may be smaller in pigs than in broiler chickens (Rodehutscord et al., 2022).

### Microbial composition

In the present study, the microbial community composition in the feces and distal ileum digesta was not affected by the treatment. Bacterial communities may stabilize in response to dietary changes (Metzler-Zebeli et al., 2013). However, Tilocca et al. (2017) found that restructuring a bacterial community after a change in diet takes several weeks. Wood and Clark (1988) reported that excess P could be stored in bacterial cells in the form of polyphosphates and be used as an energy source. Therefore, it cannot be ruled out that the adaptation period in the present study was not long enough for the microbiota to adapt, which contributed to the absence of any measured treatment effects on the microbiota composition.

Consistent with other studies (Looft et al., 2014; Zhao et al., 2015; Gierse et al., 2020), the composition of the microbial community in the distal ileum digesta was different from that in the feces. This can be attributed to the various functions and environments that exist in intestinal sections. While most digestion and absorption occur in the small intestine, in the large intestine, it is mainly the formation of VFA by fermentation. The abundance of *Lactobacillus* was lower in the distal ileum digesta than in the feces, whereas that of *Bifidobacterium* was higher. Yang et al. (2020) identified *Bifidobacterium* as the genus with the highest abundance in the ileum and colon. Metzler-Zebeli et al. (2019) found a relationship between the abundance of lactic acid-producing groups in feces and the supply of resistant starch. However, starch entry into the large intestine was not likely to differ in the present study when pc GE digestibility was used as an indicator.

*Megasphaera* correlated positively with fecal GE digestibility. *Megaspahera elsdenii* belongs to the lactate-utilizing bacterial group and can metabolize lactate as an intermediate product of carbohydrate fermentation in the hindgut (Tsukahara et al., 2002; Hashizume et al., 2003). Corn was the ingredient with the greatest proportion in all diets, and the hindgut microbiota uses resistant starch as a substrate for fermentation (Englyst et al., 1992). The genus *Megasphaeara* may utilize lactate derived from fermentation as a substrate.

*Prevotella* correlated positively with isobutyric, butyric, isovaleric, and valeric acid concentrations in feces. *Prevotella* produces acetate, which other bacteria can use to produce butyric acid (Kovatcheva-Datchary et al., 2015; Bernad-Roche et al., 2021). The positive correlation between *Bifidobacterium* and isovaleric acid concentration in feces might be due to the glycolytic abilities attributed to this genus, which generates peptidase to facilitate the utilization of N sources (Schell et al., 2002).

## Conclusion

Negative effects of dietary Ca concentration on pc InsP_6_ disappearance depend on exogenous phytase. These results imply that dietary Ca concentration must be precisely adjusted in feed formulations to avoid metabolic P loss or decreased pc InsP_6_–P release in low-P, corn-based diets with exogenous phytase. The variation in dietary Ca and the presence of exogenous phytase did not directly alter the microbial composition of the ileum and feces in the study period.

## Supplementary Material

skad254_suppl_Supplementary_Material

## Abbreviations

AA: amino acid
ALP: alkaline phosphatases
ASV: amplicon sequence variant
BUN: blood urea nitrogen
FTU: phytase unit
GE: gross energy
InsP: inositol phosphate
InsP_6_: *myo*-inositol 1,2,3,4,5,6-hexakis (dihydrogen phosphate)
N: nitrogen
Neu5Ac: *N*-acetylneuraminic acid
NMDS: non-metric multidimensional scaling
pc: prececal
PERMANOVA: permutation analysis of variance
P_i_: inorganic phosphorus
VFA: volatile fatty acid
