# Supplementary material for: Effect of dietary calcium concentration and exogenous phytase on inositol phosphate degradation, mineral digestibility, and gut microbiota in growing pigs
Source: J Anim Sci. 2023 Aug 1;101:skad254. doi: 10.1093/jas/skad254 (PMC10464513; doi:10.1093/jas/skad254)
Supplement: skad254_suppl_Supplementary_Material [file skad254_suppl_supplementary_material.docx]

**SUPPLEMENTARY MATERIAL**

of the article

**Effect of dietary calcium concentration and exogenous phytase on inositol phosphate degradation, mineral digestibility, and gut microbiota in growing pigs**

Nicolas Klein^*^, Naomi Sarpong^*^, Tanja Melzer^†^, Dieter Feuerstein^‡^, Charlotte M. E. Heyer^*^, Amélia Camarinha-Silva^*^, and Markus Rodehutscord^*^

^*^Institute of Animal Science, University of Hohenheim, 70599 Stuttgart, Germany

^†^Core Facility Hohenheim, University of Hohenheim, 70599 Stuttgart, Germany

^‡^BASF SE, 67063 Ludwigshafen, Germany

**SUPPLEMENTARY TABLES**

**Table S1** Analyzed chemical composition of the main ingredients used in the diets (g/kg DM)

| Item | Corn | Soybean meal | Rapeseed meal |
| --- | --- | --- | --- |
| DM, g/kg | 938 | 915 | 932 |
| CP (nitrogen × 6.25) | 98 | 507 | 388 |
| Ether extract | 48 | 25 | 46 |
| aNDFom^1^ | 87 | 143 | 303 |
| ADFom^2^ | 30 | 60 | 217 |
| ADL^3^ | 7 | 19 | 86 |
| Crude fiber | 20 | 46 | 133 |
| Crude ash | 13 | 76 | 83 |
| Calcium | <0.5 | 3.8 | 8.4 |
| Total P | 2.9 | 7,1 | 12.4 |
| InsP_6_-P | 2.1 | 3.7 | 5.9 |
| InsP_6,_ µmol/g DM^4^ | 11.4 | 20.0 | 31.5 |
| Ins(1,2,4,5,6)P_5_, µmol/g DM | 0.2 | 1.6 | 2.8 |
| Ins(1,2,3,4,5)P_5_, µmol/g DM | – | 0.6 | 1.8 |
| *Myo*-inositol, µmol/g DM | 1.1 | 2.2 | 1.7 |
| Phytase activity, FTU/kg | <50 | <50 | <50 |

^1^aNDFom, neutral detergent fiber assayed with heat-stable amylase and expressed exclusive of residual ash

^2^ADFom, acid detergent fiber expressed exclusive of residual ash

^3^ADL, acid detergent lignin

^4^Inositol phosphate (InsP) isomers not mentioned in the table were not detectable or were below the limit of quantification

**Table S2** Analyzed amino acid concentrations of the diets fed to growing pigs (g/kg DM)

| Dietary Ca, g/kg DM |  | 5.5 | |  | 8.5 | |
| --- | --- | --- | --- | --- | --- | --- |
| Exogenous phytase, FTU/kg of diet |  | 0 | 1,500 |  | 0 | 1,500 |
| Indispensable amino acids |  |  |  |  |  |  |
| Arg |  | 15.2 | 15.0 |  | 15.0 | 14.3 |
| His |  | 6.8 | 6.8 |  | 6.7 | 6.4 |
| Ile |  | 10.0 | 9.9 |  | 9.8 | 9.3 |
| Leu |  | 21.1 | 20.9 |  | 21.0 | 20.0 |
| Lys |  | 12.6 | 12.6 |  | 12.5 | 11.7 |
| Met |  | 4.0 | 4.0 |  | 4.0 | 3.9 |
| Phe |  | 11.7 | 11.5 |  | 11.6 | 11.0 |
| Thr |  | 9.6 | 9.6 |  | 9.5 | 9.0 |
| Val |  | 11.5 | 11.4 |  | 11.4 | 10.7 |
| Dispensable amino acids |  |  |  |  |  |  |
| Ala |  | 12.6 | 12.5 |  | 13.2 | 11.8 |
| Asx^1^ |  | 23.0 | 22.8 |  | 22.7 | 21.8 |
| Cys |  | 4.2 | 4.3 |  | 4.2 | 3.9 |
| Glx^1^ |  | 43.4 | 43.3 |  | 43.3 | 41.5 |
| Gly |  | 10.4 | 10.4 |  | 10.5 | 9.7 |
| Pro |  | 14.7 | 14.7 |  | 14.8 | 14.1 |
| Ser |  | 12.0 | 12.0 |  | 12.0 | 11.5 |
| Tyr |  | 8.0 | 7.9 |  | 7.9 | 7.5 |

^1^Asp and Asn, Glu and Gln, respectively, could not be distinguished because the Asn and Gln side groups were lost during acid hydrolysis (Fontaine, 2003)

**Table S3** Prececal amino acid (AA) digestibility of experimental diets fed to growing pigs^1^

| Dietary Ca, g/kg DM |  | 5.5 | |  | 8.5 | | SEM | *P*-value | | |
| --- | --- | --- | --- | --- | --- | --- | --- | --- | --- | --- |
| Exogenous phytase, FTU/kg of diet |  | 0 | 1,500 |  | 0 | 1,500 |  | Ca | Phytase | Ca × Phytase |
| Indispensable AA, % |  |  |  |  |  |  |  |  |  |  |
| Arg |  | 87.7 | 88.5 |  | 87.5 | 87.6 | 0.42 | 0.149 | 0.250 | 0.400 |
| His |  | 81.1 | 81.3 |  | 80.9 | 79.7 | 0.62 | 0.129 | 0.452 | 0.254 |
| Ile |  | 82.7 | 83.4 |  | 82.0 | 81.8 | 0.68 | 0.092 | 0.655 | 0.492 |
| Leu |  | 83.1 | 83.6 |  | 82.7 | 82.2 | 0.72 | 0.176 | 0.913 | 0.465 |
| Lys |  | 81.5 | 82.2 |  | 80.8 | 80.7 | 0.64 | 0.100 | 0.605 | 0.580 |
| Met |  | 85.1 | 85.5 |  | 85.1 | 84.4 | 0.63 | 0.356 | 0.942 | 0.357 |
| Phe |  | 83.6 | 84.3 |  | 83.1 | 83.0 | 0.64 | 0.144 | 0.502 | 0.547 |
| Thr |  | 72.9 | 73.6 |  | 72.2 | 71.1 | 0.98 | 0.087 | 0.852 | 0.344 |
| Val |  | 79.6 | 80.2 |  | 79.2 | 78.3 | 0.76 | 0.123 | 0.912 | 0.337 |
| Dispensable AA, % |  |  |  |  |  |  |  |  |  |  |
| Ala |  | 78.9 | 79.3 |  | 79.2 | 77.2 | 0.82 | 0.255 | 0.352 | 0.149 |
| Asx^2^ |  | 78.1 | 79.2 |  | 77.8 | 77.8 | 0.65 | 0.180 | 0.332 | 0.380 |
| Cys |  | 72.5 | 73.3 |  | 72.8 | 70.8 | 1.17 | 0.234 | 0.530 | 0.155 |
| Glx^2^ |  | 84.4 | 85.4 |  | 84.5 | 84.5 | 0.65 | 0.489 | 0.428 | 0.425 |
| Gly |  | 70.4 | 70.9 |  | 69.2 | 68.6 | 1.83 | 0.111 | 0.886 | 0.547 |
| Pro |  | 77.7 | 78.4 |  | 77.4 | 77.5 | 0.94 | 0.378 | 0.579 | 0.722 |
| Ser |  | 78.2 | 79.5 |  | 78.5 | 77.8 | 0.72 | 0.335 | 0.664 | 0.181 |
| Tyr |  | 82.2 | 82.5 |  | 81.6 | 81.1 | 0.74 | 0.151 | 0.995 | 0.651 |

^1^Least squares means based on eight observations per diet

^2^Asp, Asn and Glu, Gln, respectively, were not distinguished because the Asn and Gln side groups were lost during acid hydrolysis (Fontaine, 2003)

**Table S4** Volatile fatty acid (VFA) concentration of feces^1^ (mmol/kg, wet basis)

| Dietary Ca, g/kg DM |  | 5.5 | |  | 8.5 | | SEM | *P*-value | | |
| --- | --- | --- | --- | --- | --- | --- | --- | --- | --- | --- |
| Exogenous phytase, FTU/kg of diet |  | 0 | 1,500 |  | 0 | 1,500 |  | Ca | Phytase | Ca × Phytase |
| Acetic acid |  | 50.6 | 53.2 |  | 59.2 | 56.7 | 3.75 | 0.059 | 0.872 | 0.298 |
| Propionic acid |  | 22.1 | 22.8 |  | 23.4 | 22.9 | 1.89 | 0.662 | 0.990 | 0.547 |
| Iso-butyric acid |  | 3.3 | 3.5 |  | 3.9 | 3.8 | 0.33 | 0.037 | 0.559 | 0.401 |
| Butyric acid |  | 18.2 | 18.6 |  | 19.6 | 17.7 | 1.90 | 0.615 | 0.744 | 0.175 |
| Iso-valeric acid |  | 5.8 | 6.3 |  | 7.0 | 6.7 | 0.69 | 0.020 | 0.461 | 0.187 |
| Valeric acid |  | 6.8 | 7.2 |  | 6.9 | 7.0 | 1.10 | 0.562 | 0.263 | 0.712 |

^1^Means based on eight observations per diet

**Table S5** Output of permutation analysis of variance (PERMANOVA) for ileal digesta samples

|  | DF | SS | R-squared | Pseudo-F | *P*-value |
| --- | --- | --- | --- | --- | --- |
| Phytase | 1 | 0.05 | 0.01 | 0.27 | 0.96 |
| Ca | 1 | 0.03 | 0.01 | 0.18 | 0.99 |
| Ca × Phytase | 1 | 0.11 | 0.02 | 0.61 | 0.72 |
| Residual | 28 | 5.19 | 0.96 |  |  |
| Total | 31 | 5.39 | 1 |  |  |

DF, degree of freedom

SS, sum of squares

**Table S6** Output of permutation analysis of variance (PERMANOVA) for fecal samples

|  | DF | SS | R-squared | Pseudo-F | *P*-value |
| --- | --- | --- | --- | --- | --- |
| Phytase | 1 | 0.18 | 0.03 | 0.99 | 0.39 |
| Ca | 1 | 0.09 | 0.02 | 0.47 | 0.99 |
| Ca × Phytase | 1 | 0.26 | 0.05 | 1.42 | 0.09 |
| Residual | 27 | 4.98 | 0.90 |  |  |
| Total | 30 | 5.51 | 1 |  |  |

DF, degree of freedom

SS, sum of squares

**Table S7** Output of permutation analysis of variance (PERMANOVA) for fecal and ileal digesta samples

|  | DF | SS | R-squared | Pseudo-F | *P*-value |
| --- | --- | --- | --- | --- | --- |
| Sample type | 1 | 2.7846 | 0.20351 | 15.586 | <0.001 |
| Residual | 61 | 10.8979 | 0.79649 |  |  |
| Total | 62 | 13.6825 | 1 |  |  |

DF, degree of freedom

SS, sum of squares

**Table S8** Significant *P-*values for the comparison of microbiota of ileal digesta and fecal samples at the genus level

| Genus | *P*-value^1^ |
| --- | --- |
| *Clostridium sensu stricto* | <0.001 |
| *Lactobacillus* | <0.001 |
| *Limosilactobacillus* | 0.016 |
| *Olsenella* | <0.001 |
| *Prevotella* | <0.001 |
| *Streptococcus* | 0.002 |
| *Terrisporobacter* | 0.022 |
| Unclassified *Prevotellaceae* | <0.001 |
| Unclassified *Ruminococcaceae* | 0.048 |

^1^Benjamini–Hochberg adjusted *P*-values

**SUPPLEMENTARY FIGURE**


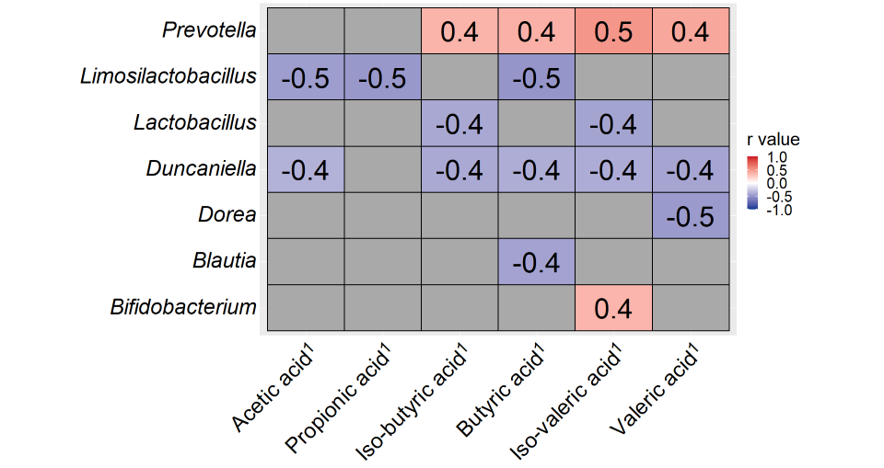


**Figure S1** Significant correlations (*P* < 0.05) between relative abundance of bacterial groups and volatile fatty acid concentrations in the feces. ^1^mmol/kg of feces

**Literature cited in the supplementary material**

Fontaine, J. 2003. Amino acid analysis of feeds. In: D’Mello, J. P. F., editor. Amino acids in animal nutrition. 2nd ed. Wallingford, UK and Cambridge. CABI Publishers; p. 15–40.
